# Unraveling the Hidden Potential of Barley (*Hordeum vulgare*): An Important Review

**DOI:** 10.3390/plants13172421

**Published:** 2024-08-30

**Authors:** Avneet Kaur, Sukhvinder Singh Purewal, Yuthana Phimolsiripol, Sneh Punia Bangar

**Affiliations:** 1 Department of Chemistry, University Institute of Sciences, Chandigarh University, Mohali 140413, Punjab, India; avneet.e13480@cumail.in; 2 University Centre for Research & Development (UCRD), Chandigarh University, Mohali 140413, Punjab, India; sukhvinder.e14148@cumail.in; 3 Faculty of Agro-Industry, Chiang Mai University, Chiang Mai 50100, Thailand; yuthana.p@cmu.ac.th; 4 Department of Food, Nutrition and Packaging Sciences, Clemson University, Clemson, SC 29634, USA

**Keywords:** barley, bioactive profile, food, nutritional composition

## Abstract

Barley (*Hordeum vulgare*) is a winter crop well known for its small-seeded grains and self-pollinating characteristics. The flour derived from barley grains plays a crucial role in numerous processed food items, contributing to their taste and nutritional value. Barley consists of complex carbohydrates (80%), proteins (11.5–14.2%), lipids (4.7–6.8%), β-glucans (3.7–7.7%), and ash (1.8–2.4%). Beyond its other nutrients, barley boasts a good reservoir of phenolic compounds (1.2–2.9 mg/g GAE). This abundance of beneficial compounds positions barley as an attractive industrial substrate. In this review, the nutritional composition and bioactive profile of barley are discussed in a systemic manner, emphasizing its potential in the development of innovative barley-based products that promote health and well-being. By incorporating barley into various food formulations, industries can not only boost nutritional content but also offer consumers a wide range of health benefits. In conclusion, barley’s diverse applications in food and health highlight its essential role in promoting healthier living.

## 1. Introduction

Barley (*Hordeum vulgare*) stands as an important grain crop among cereal grains for its remarkable nutritional profile. Barley grains are characterized by a unique blend of macronutrients, micronutrients, and bioactive compounds [1]. Macronutrients such as carbohydrates, the primary energy source that fuels bodily functions, are inherent elements of barley grains. A unique blend of macro- and micronutrients makes barley an important industrial substrate for commercial usage [2,3,4]. Barley consists of complex carbohydrates (80%), proteins (11.5–14.2%), lipids (4.7–6.8%), β-glucan (3.7–7.7%), and ash (1.8–2.4%). Starch is the most prevalent nutrient in barley, accounting for 70% of the kernel’s total dry weight [5,6,7]. Soluble fibers, particularly β-glucan, offer a symphony of cardiovascular benefits, while insoluble fibers uphold the fortifications of digestive health. The growing interest in natural antioxidants is driven by the remarkable radical scavenging potential exhibited by phytochemicals, particularly phenolic compounds, flavonoids, and tannins, abundant in barley [8,9,10,11]. Recent scientific research underscores the necessity of a synergistic blend of phytochemicals sourced from cereal grains to counteract the detrimental effects of free radicals, implicated in various diseases [12,13,14,15,16,17,18]. Barley flour, whether processed or unprocessed, finds promising applications as a substitute for wheat flour in select functional foods, bakery products, and household recipes. This review paper helps to elucidate the nutritional composition along with bioactive phytochemicals and health-benefiting potential of barley.

## 2. Barley Production Status

**Figure 1:**
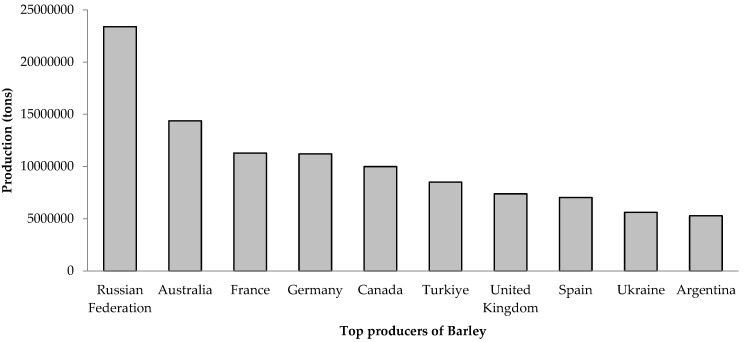
Worldwide top barley producers during 2022 [19].

The data regarding the area under cultivation, production, and yield of barley in India and across the globe from 2012 to 2022 were obtained from the Food and Agriculture Organization (FAO) in 2024, as outlined in Table 1 and Table 2 [19]. Leading the global production figures are countries such as the Russian Federation, Australia, France, Germany, and Canada (Figure 1). The maximum global barley production was reached in 2019, totaling 158,827,743 tons, while the lowest was observed in 2012, with a production of 132,412,417 tons. The fluctuation in global barley production indicates the influence of various factors, including climatic conditions, market demand, and technological advancements in agriculture. In India, barley production peaked in 2014 at 1,830,000 tons but declined to its lowest point of 1,371,360 tons by 2022. This decrease can be attributed to changes in agricultural practices, shifts in farmers’ crop preferences, and climatic challenges. While global barley production experienced a notable peak in 2019, India’s production followed a declining trend. This divergence suggests that local factors in India, such as policy changes, market dynamics, and regional climatic conditions, significantly impact barley production independently of global trends. India has the potential to increase its barley production, but achieving this requires addressing specific local challenges and learning from global best practices. By enhancing agricultural support, adopting advanced technologies, and developing climate-resilient strategies, India can potentially reverse the declining trend in barley production and contribute more significantly to the global market.

Barley production in specific countries is shaped by multiple factors, including climatic suitability, advanced agricultural methodologies, economic incentives, and market demand [20,21,22]. Barley thrives in cooler climates, making it an ideal crop for temperate regions like Canada, Russia, and parts of Europe, where climatic conditions are optimal for its cultivation. Countries with advanced agricultural systems, employing modern machinery, efficient irrigation, and innovative crop management strategies, often achieve superior barley yields. This is particularly evident in nations like Germany, France, and Canada. Additionally, barley’s role in crop rotation systems enhances soil fertility and helps manage weeds and pests, further boosting production in areas with sustainable agricultural practices. Government interventions, such as subsidies and supportive policies, make barley cultivation more appealing to farmers, especially in parts of the European Union and Canada. Proximity to major markets and robust export infrastructure also significantly contribute to higher barley production. European nations, in particular, benefit from their strategic location near high-demand markets, facilitating increased production and distribution. Moreover, countries with strong agricultural research capabilities, such as Australia and Canada, invest in developing high-yield, disease-resistant barley varieties. Well-established export markets for barley and its derivative products stimulate continuous production growth to satisfy international demand, solidifying these countries’ positions as leading barley producers [20]. Continuous cultivation without proper soil management leads to degradation, reduced fertility, and lower yields. To counteract this, implementing sustainable practices like crop rotation, cover cropping, and the use of organic amendments is essential for restoring soil health [23,24,25]. Economic factors such as fluctuating market prices and input costs can affect farmers’ willingness to invest in barley production. Establishing cooperatives and farmer networks can improve market access, while government policies and subsidies should support sustainable practices and mitigate price volatility. In water-scarce regions, promoting drought-tolerant barley varieties, efficient irrigation, and rainwater harvesting can help maintain production. Additionally, investing in transportation and storage infrastructure, along with improving market access through policy support and trade agreements, is vital for stabilizing and increasing barley production.

## 3. Nutritional Profile

Barley has an impressive nutritional profile, making it a valuable addition to a balanced diet. A unique blend of macro- and micronutrients makes it a preferable substrate for food processing industries. The nutritional profile of barley is explained in Table 3.

### 3.1. Barley Starch

Barley starch primarily comprises two polysaccharides: amylose and amylopectin. Amylose consists of linear glucose units linked by α-1,4-glycosidic bonds, whereas amylopectin is a branched polymer featuring α-1,4 and α-1,6-glycosidic linkages. The ratio of amylose to amylopectin in barley starch varies across cultivars, impacting its physicochemical characteristics [29,30,31]. Barley starch granules demonstrate a characteristic bimodal distribution, comprising A-type and B-type granules, each possessing distinct morphological and crystalline properties. A-type granules are larger and exhibit irregular shapes, while B-type granules are smaller and more spherical. Earlier published scientific studies demonstrated that, for normal starch, the yield ranges from 86.67% to 98.79%, with amylose content between 21.80% and 26.57%, and amylopectin from 64.86% to 77.88% [32,33,34,35,36,37]. The starch granules exhibit a variety of shapes, including oval, cuboidal, and irregular, and their sizes range from 6.96 µm to 30 µm. The X-ray pattern is predominantly A-type, with relative crystallinity between 22.30% and 43.21%. For waxy starch, the yield is between 58.1% and 68.5%, with an extremely high amylose content of 99.81% to 99.39%. The amylopectin content ranges from 0% to 4.5%. The starch granules typically have large pores, and the X-ray pattern remains predominantly A-type [38,39]. High-amylose starch is also mentioned, with yield percentages between 59.7% and 61.9%, amylose content from 38.4% to 44.1%, and amylopectin from 27.74% to 31.04%. The granules often exhibit small pores, with A- and B-type X-ray patterns [40,41]. The unique structural features of barley starch influence its functional properties and applications. Barley starch exhibits unique physicochemical properties, including granule size, morphology, gelatinization, retrogradation, viscosity, pasting behavior, and digestibility. Granule size distribution and morphology affect starch functionality in food and industrial applications [42,43,44,45]. The viscosity and pasting behavior of barley starch influence its suitability for various food formulations and processing methods. Starch modification techniques include physical treatments (e.g., heat–moisture treatment and annealing), chemical modifications (e.g., cross-linking, esterification, and oxidation), and enzymatic modifications (e.g., hydrolysis, transglycosylation, and solid-state fermentation) [46,47,48]. Barley starch finds diverse applications in food and non-food industries due to its unique properties and functionalities. In the food industry, barley starch is used as a thickening agent, gelling agent, stabilizer, and texturizer in various products such as bread, noodles, soups, sauces, desserts, and beverages. It provides texture, mouthfeel, and shelf stability to food formulations and contributes to product quality and sensory attributes. In the non-food sector, barley starch is utilized in pharmaceuticals for tablet binding and coating, in papermaking for paper sizing and coating, and in bioethanol production as a renewable feedstock for fermentation. The versatility and sustainability of barley starch make it an attractive ingredient for a wide range of applications, contributing to the economic value and environmental benefits of barley cultivation [49,50].

### 3.2. Protein

Barley protein is composed of different fractions, including albumins, globulins, prolamins (gluten), and glutelins, each with unique properties and functionalities. Albumins and globulins are soluble proteins found in barley grains, while prolamins and glutelins are insoluble proteins that form the gluten matrix. The functional properties of barley protein, including solubility, gelation, emulsification, foaming, and water-holding capacity, are influenced by its structural characteristics, such as molecular weight distribution, secondary structure, and surface hydrophobicity. These functional properties make barley protein suitable for a wide range of food and non-food applications [51,52]. Protein content in barley ranges from 8% to 13%, synthesized during grain development in the endosperm and aleurone layer. Hordein, the main storage protein, constitutes 40–50% of barley’s protein and is rich in glutamine and proline. Glutelin, the second most abundant protein, comprises 35–45% of the total storage protein [53,54,55]. Protein Z, a significant barley albumin and member of the serpin protein group, accounts for about 5% of the albumin content. The protein content in barley is critical for assessing malting and brewing quality. Higher protein levels result in lower carbohydrate content, reducing extract yield and enzyme activity. Conversely, lower protein levels can negatively affect yeast nutrition, enzymatic activity, and fermentable sugar production, also leading to poor extract yield. Variability in barley protein content is influenced by factors such as cultivar, environmental conditions, and fertilizer use. Barley, used in both raw and malted forms, is often blended with rice and corn [56]. Barley contains prolamin storage proteins called hordeins, which vary by molecular weight and location. A-hordeins have a molecular weight of 12–26 kDa, B-hordeins range from 36 to 45 kDa, C-hordeins range from 59 to 72 kDa, and D-hordeins have a molecular weight of approximately 100 kDa [57,58,59,60]. Barley protein has diverse applications in various industries, including food, animal feed, pharmaceuticals, cosmetics, and bioplastics. In the food industry, barley protein is used as a functional ingredient in products such as bread, pasta, cereal bars, beverages, and meat analogs. It provides texture, structure, and nutritional value to food formulations and contributes to product quality and sensory attributes. In the animal feed industry, barley protein is utilized as a protein source in feed formulations for livestock, poultry, and aquaculture. It serves as a sustainable alternative to traditional protein sources such as soybean meal and fishmeal. In the pharmaceutical and cosmetic industries, barley protein is used in the formulation of pharmaceuticals, skincare products, and haircare products due to its moisturizing, emollient, and film-forming properties [54,61]. Barley protein is being explored as a renewable and biodegradable material for the production of bioplastics, packaging materials, and biomaterials. Research and innovation in processing technologies, product development, and market expansion are essential to maximize the utilization of barley protein and enhance its value as a sustainable protein source in a rapidly growing global market. Understanding the structure, composition, functionality, and applications of barley protein is crucial for optimizing its utilization and meeting the increasing demand for nutritious and sustainable protein sources [62].

### 3.3. Fibers

Barley fibers consist of both insoluble fibers (cellulose, hemicellulose, and lignin) and soluble fibers (β-glucans). Insoluble fibers contribute to fecal bulking and promote regular bowel movements, while soluble fibers form viscous gels in the gastrointestinal tract, slowing down digestion and absorption of nutrients [63,64,65]. Barley β-glucans, a type of soluble fiber, have garnered significant attention due to their cholesterol-lowering and immunomodulatory effects. Barley fibers possess unique physicochemical properties, including water-holding capacity, swelling capacity, viscosity, and fermentability [66,67]. Barley fibers are resistant to enzymatic digestion in the small intestine and are fermented by beneficial gut microbiota in the colon, producing short-chain fatty acids and other metabolites with health-promoting effects. The consumption of barley fibers has been associated with numerous health benefits, including improved digestion, weight management, glycemic control, and cardiovascular health. Barley fibers promote satiety and reduce energy intake, thereby aiding in weight management and obesity prevention. They also modulate blood glucose levels and insulin sensitivity, making them beneficial for individuals with diabetes or at risk of developing diabetes [2,68,69,70]. In the dietary supplement industry, barley fibers are utilized in the formulation of fiber supplements, prebiotics, and digestive health products. Barley fibers are valuable and versatile ingredients with significant health benefits and diverse applications in food and non-food industries. Their unique physicochemical properties and physiological effects make them ideal for promoting human health and sustainability. Further research and innovation in processing technologies, product development, and market expansion are essential to maximize the utilization of barley fibers and enhance their value as functional and sustainable ingredients in a rapidly evolving global market [71,72]. Barley β-glucan is a soluble dietary fiber that has gained widespread attention for its health-promoting properties. It is a polysaccharide composed of glucose units linked together by beta-glycosidic bonds. It has a linear backbone with occasional branching, resulting in a complex three-dimensional structure. The molecular weight, degree of branching, and solubility of barley β-glucan can vary depending on factors such as barley variety, processing methods, and extraction techniques. Barley β-glucan exhibits a range of physiological effects in the human body, primarily due to its unique structural properties. As a soluble fiber, barley β-glucan forms viscous gels in the gastrointestinal tract, which can delay gastric emptying, slow glucose absorption, and reduce cholesterol reabsorption. Additionally, barley β-glucan acts as a prebiotic, promoting the growth of beneficial gut bacteria and improving gut health [65,73,74]. It acts as a prebiotic, providing fermentable substrates for beneficial gut bacteria, which produce short-chain fatty acids and other metabolites with health-promoting effects [75,76,77,78].

### 3.4. Vitamins and Minerals

Barley is a good source of essential vitamins, including B-vitamins (thiamine, riboflavin, niacin, pantothenic acid, pyridoxine, biotin, folate, and cobalamin), vitamin E, and vitamin K. Vitamin E is a potent antioxidant that protects cells from oxidative damage, while vitamin K plays a crucial role in blood clotting and bone metabolism [3]. Barley is an important cereal grain that possesses different tocols, especially α-tocotrienols and α-tocopherols. Vitamin E content in barley ranges from 8.5 to 31.5 µg/g dry weight and is influenced by both storage conditions and genotype. Lower Vitamin E levels are often found in genotypes with pigmentation or hulls [79,80]. The highest concentrations of tocols are located in the endosperm, hull, and germ of barley, with levels of 95%, 63%, and 10%, respectively [81]. Barley is also rich in essential minerals, including potassium, magnesium, phosphorus, calcium, iron, zinc, copper, manganese, and selenium. These minerals are involved in numerous physiological processes, such as muscle contraction, nerve transmission, bone formation, blood clotting, oxygen transport, enzyme activation, and immune function [82]. The consumption of barley grass, rich in vitamins and minerals, may help prevent nutrient deficiencies and reduce the risk of chronic diseases such as cardiovascular disease, osteoporosis, and cancer [83].

### 3.5. Bioactive Phytochemicals

The term “bioactive phytochemicals” refers to a combination of active components found naturally, each with solubility specific to the solvent used [84,85]. Beyond its role as a dietary staple, barley is recognized for its rich content of bioactive compounds, which contribute to its nutritional value and health-promoting antioxidant properties. These include hydroxybenzoic acids (e.g., gallic acid), hydroxycinnamic acids (e.g., ferulic acid), flavonoids (e.g., quercetin and kaempferol), and lignans. Barley contains a diverse range of flavonoids, including flavonols, flavones, flavanones, and anthocyanins. These compounds exhibit antioxidant, anti-inflammatory, antimicrobial, and anticancer properties. Table 4 represents TPC values in barley extracts, and Table 5 demonstrates the pharmaceutical potential of barley phytochemicals. Barley grains, along with their bran and husk components, contain a variety of bioactive phytochemicals, including total phenolic compounds (TPC), condensed tannin content (CTC), flavonoids, tocopherols, and alkylresorcinols [1,86,87,88]. The reported TPC levels in barley cultivars range from 1.95 to 2.20 mg GAE/g dwb [89], 2.09 to 2.94 mg GAE/dwb [1], and 3.07 to 4.43 mg FAE/g [90]. Madhujith and Shahidi [91] found TPC values ranging from 2.63 to 4.51 mg FAE/g. The composition of TPC in barley may vary due to factors such as temperature, pH, soil type, moisture content, fertilizer use, and harvest stage. Additionally, barley grains contain flavanols such as myricetin, catechin, and procyanidin B3 [92]. Sharma and Gujral [86] reported total flavonoid content (TFC) in barley ranging from 1.38 to 2.24 mg CE/g dwb. CTC in barley cultivars ranges from 0.40 to 0.99 mg CE/g dwb [1], while Collins [93] reported 0.74 mg/g tannins in barley. Specific bioactive phytochemicals in barley have been identified using techniques such as HPLC and HPTLC, revealing the presence of gallic acid, ferulic acid, o-hydroxybenzoic acid, vanillic acid, syringic acid, p-coumaric acid, cinnamic acid, and caffeic acid [16,94,95,96,97,98,99].

## 4. Barley-Based Food Products

**Figure 2:**
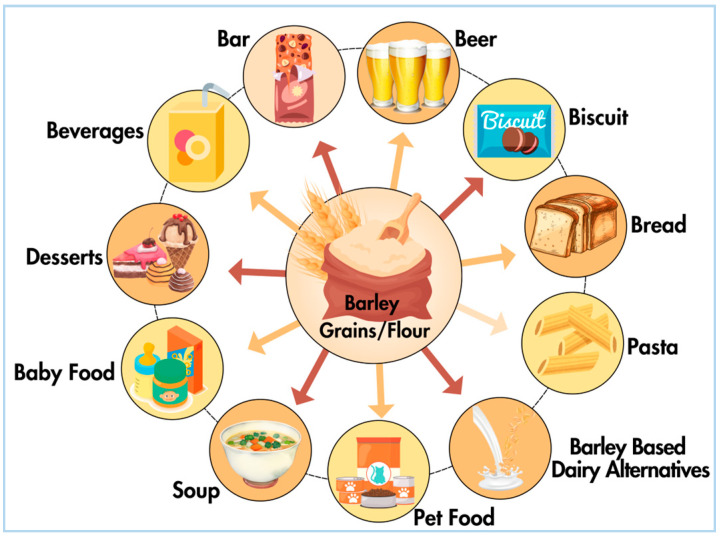
Different barley-based food products.

Barley-based bread is a popular food product made from barley flour or barley-based blends (Figure 2). It offers a nutritious alternative to traditional wheat bread, with higher fiber content, lower glycemic index, and enhanced flavor profile. Barley-based pasta is another popular food product that offers a nutritious alternative to traditional wheat pasta. It is typically made from a blend of barley flour and durum wheat semolina, providing a unique texture and flavor profile. Barley-based pasta is rich in dietary fiber, protein, vitamins, and minerals, making it a nutritious choice for pasta lovers seeking healthier options [121,122]. This product category includes a variety of shapes and forms, such as spaghetti, penne, fusilli, and lasagna, catering to diverse culinary preferences and dietary needs. Barley-based cereal bars and snacks are convenient and portable food products that offer a nutritious and satisfying snack option for on-the-go consumption [62]. Barley-based beverages encompass a wide range of products, including barley water, barley tea, barley coffee substitutes, and barley-based milk alternatives [123]. Barley-based dairy alternatives, such as barley milk and barley yogurt, are emerging as plant-based alternatives to traditional dairy products. These products are made from barley grains, barley malt extract, or barley flour, combined with water, emulsifiers, stabilizers, and flavorings [62]. Fermentation enhances the flavor, aroma, and nutritional value of barley-based foods while also promoting the growth of beneficial bacteria and enzymes. These fermented products offer unique culinary experiences and potential health benefits associated with probiotics and bioactive compounds. Barley can be processed into a variety of snack foods, including roasted barley snacks, popped barley chips, and barley crackers. These snacks offer a crunchy texture, savory flavor, and nutritional benefits, making them a popular choice for guilt-free snacking [124,125]. Barley-based snack foods are often marketed as gluten-free, whole grain options, appealing to health-conscious consumers looking for convenient and satisfying snacks. Barley is also used as an ingredient in pet foods, including dog and cat kibble, treats, and supplements. It provides carbohydrates, fiber, protein, vitamins, and minerals to support the nutritional needs of pets [126]. Barley-based pet foods may be formulated for specific life stages, dietary preferences, or health conditions, catering to the diverse needs of companion animals. Fermented barley could also be used in the preparation of antioxidant-rich food supplements [127]. The future scope and novel uses of barley are presented in Table 6.

## 5. Health Benefits of Barley

Several studies have demonstrated that the regular consumption of barley or barley-derived products can lower the risk of cardiovascular disease, hypertension, and stroke. Barley arabinoxylan has been investigated for its potential to regulate lipid and glucose metabolism and promote digestive health [128,129,130]. Recent research focused on its hypoglycemic effects in mice, showing that a single dose of arabinoxylan extract followed by a glucose solution resulted in suppressed blood glucose elevation over 12 weeks [129]. Arabinoxylan, alongside β-glucan, was found to enhance GLP-1 secretion mediated by SCFAs upon barley consumption, suggesting its role in managing type 2 diabetes [130]. Soluble fibers derived from barley, when fed to rats with type 2 diabetes, effectively lowered postprandial blood glucose, improved insulin sensitivity, and reduced body weight [63]. The presence of propionic acid, linked to improved glucose regulation and insulin response, was attributed to the promotion of Akkermansia growth by soluble arabinoxylan [131]. Additionally, arabinoxylan and β-glucan were found to promote the growth of butyrate-producing bacteria, contributing to enhanced gut microbiota diversity compared to purer fiber extracts [132]. These fibers also exhibited beneficial effects on liver function, increasing HDL cholesterol, and decreasing serum malondialdehyde levels, indicating potential antioxidant and cardioprotective properties, similar to those observed with β-glucan intake [130]. Arabinoxylan extracted from brewer’s spent grain demonstrated notable prebiotic effects by increasing levels of beneficial gut bacteria, particularly Lactobacillus and Bifidobacteria [131]. Regarding its food applications, barley arabinoxylan shows promise as a versatile ingredient across various food products. In bread and bakery items, it can act as a viscosity modulator, enhance structure, and reduce the glycemic index, catering to individuals with diabetes [129]. It can also facilitate the production of low-calorie, high-fiber bread and pasta products and aid in fat and sugar reduction due to its bulk effect and binding capacity [133,134]. Moreover, barley arabinoxylan enhances the texture and satisfaction of cereal-based snacks, reduces fat content, and can be incorporated into functional foods like snack bars for increased fiber content and enhanced satiety [135,136]. In sports drinks and supplements, it can be formulated into concentrated fiber shots or blended with other ingredients to meet specific dietary needs for athletes. Additionally, it finds applications in ready-to-eat meals, dairy products, and processed meats, where it improves texture, stability, and moisture retention while promoting gut health [137].

Animal experiments and clinical trials have provided additional support for the potential anti-obesity effects of barley (HB) and its extracts. For instance, a study by Gong et al. [138] demonstrated that feeding germ-free obese mice a diet containing 46% whole barley for 9 weeks led to symptom alleviation, primarily by inhibiting cholesterol synthesis and enriching gut microbiota. Similarly, Deng et al. [139] found that administering whole barley grain orally improved obesity symptoms in the db/db mice model, with mechanisms involving the regulation of the AMPK/SREBP-1c/FAS pathway and fermentation by cecal microbiota. Certain cecal bacteria, such as Prevotella and Anaerovibrio, were found to produce more short-chain fatty acids (SCFAs), with their enrichment observed in obese mice consuming whole grain Qingke diet [140]. Studies have also shown that barley β-glucans have beneficial effects on fatty liver and insulin sensitivity in high-fat diet-fed mice [141]. Upon comparing a high-fat–sucrose diet made with refined wheat flour to one substituted with whole grain barley, significant reductions in fat deposits were observed in rats and mice models [138,142]. Additionally, a randomized double-blind human study by Aoe et al. [75] found that barley rich in β-glucan significantly reduced visceral fat area, BMI, and waist circumference in obese Japanese subjects over 12 weeks. Liu et al. [143] observed that barley intake reduced postprandial changes in glucose and insulin levels, potentially preventing early diabetes development. This trial indicated that barley could modulate metabolic dysregulation, possibly through the activation of the gut hormone GLP-1. Barley contains various bioactive compounds, including antioxidants, vitamins, and minerals, that have been shown to possess anticancer properties. These compounds help neutralize free radicals, inhibit the growth of cancer cells, and reduce inflammation. Studies suggest that the regular consumption of barley may lower the risk of colorectal cancer, breast cancer, and prostate cancer. Elevated blood cholesterol levels significantly increase the risk of cardiovascular disease (CVD). It is widely recognized that soluble dietary fiber, particularly β-glucan found in barley and oats, plays a crucial role in preventing heart disease [3,144]. Unlike other cereals like rice, maize, and wheat, barley is considered a good source of β-glucan. Research suggests that barley is comparable to oats in effectively lowering cholesterol levels. Delaney et al. [145] observed decreased aortic cholesterol and increased fecal neutral cholesterol in hamsters fed with 8 g/100 g β-glucan sourced from barley or oats. Soluble β-glucan, a key component found in oat or barley seeds, is believed to contribute to cholesterol reduction. It functions by increasing intestinal viscosity, thereby slowing the absorption of dietary oils. Moreover, β-glucan binds to bile acids, promoting their discharge, which stimulates the body to replace cholesterol. Tocopherol and tocotrienol have also been shown to reduce cholesterol levels, offering further benefits in preventing and treating cardiovascular disease and cancer when incorporated into healthy diets. Dietary fiber, recognized for its role in mitigating CVD risks, enhancing satiety, and improving gastrointestinal immunity, has garnered attention for its potential anticancer effects. Research on the anticancer properties of barley (HB) has mainly focused on colon and liver cancer prevention. Studies suggest that HB polysaccharides inhibit proliferation and induce the apoptosis of cancer cells, potentially through the regulation of reactive oxygen species and transcription factors. Additionally, β-glucan exhibits potent antitumor effects by interacting with receptors in macrophages and modulating immune responses. Both high- and low-molecular-weight β-glucan from barley show promise in cancer immunotherapy, with low-molecular-weight β-glucan promoting dendritic cell maturation, a crucial component of the immune system’s response to tumors [146]. Phenolic extracts from barley demonstrate antiproliferative effects on liver cancer cells, with free forms showing better activity compared to bound forms. Bioactive compounds such as GABA and tocotrienols exhibit potential anticancer properties [147,148,149,150].

## 6. Conclusions

Barley-based food products offer a diverse range of nutritious and flavorful options for consumers seeking healthier and more sustainable dietary choices. These products are rich in fiber, protein, vitamins, minerals, and antioxidants, which contribute to their nutritional value and health-promoting properties. Market trends indicate a growing demand for barley-based food products, driven by increasing consumer awareness of the health benefits of whole grains and plant-based foods, as well as growing concerns about sustainability. To fully capitalize on this potential, research must focus on optimizing barley’s nutritional profile through selective breeding and advanced processing technologies, ensuring the retention of health-promoting compounds. Effective consumer education and product characterization will further promote barley’s integration into health benefiting diverse diets.

## Figures and Tables

**Table 1:** Area, yield, and production of barley during the last ten years (2012–2022) in India [19].

Year	2012	2013	2014	2015	2016	2017	2018	2019	2020	2021	2022
Area (ha)	643,000	695,000	673,500	707,000	590,000	656,250	660,800	575,600	589,570	592,470	453,320
Yield (100 g/ha)	25,194	25,180	27,171	22,815	24,407	26,628	26,949	28,372	29,205	27,957	30,251
Production (tons)	1,620,000	1,750,000	1,830,000	1,613,000	1,440,000	1,747,450	1,780,810	1,633,070	1,721,830	1,656,340	1,371,360

**Table 2:** Worldwide area, yield, and production of barley during the last ten years (2012–2022) [19].

Year	2012	2013	2014	2015	2016	2017	2018	2019	2020	2021	2022
Area (ha)	50,049,962	50,012,050	49,931,707	49,049,425	48,207,088	47,876,909	48,057,496	51,318,477	51,959,891	48,781,497	47,147,005
Yield (100 g/ha)	26,456	28,730	29,099	30,249	30,307	31,014	29,343	30,949	30,184	29,752	32,850
Production (tons)	132,412,417	143,683,812	145,294,109	148,370,300	146,101,933	148,486,476	141,013,838	158,827,743	156,836,485	145,133,126	154,877,139

**Table 3:** Different nutrient components in barley.

Nutrient	Concentration	References
Starch	50.8–56.3%	[26,27]
Crude fat	1.6–2.63%	
Crude protein	11.5–16.2%	
Crude fiber	11.7–16.5%	
Lipids	2–3%	
Sodium	122–146 mg/kg	
Calcium	312–368 mg/kg	
Magnesium	527–616 mg/kg	
Iron	43–66 mg/kg	
Zinc	22.5–26.6 mg/kg	
Histidine	2.6–3.2 (g/100 g protein)	
Threonine	3.2–3.7 (g/100 g protein)	
Phenylalanine	6.7–9.2 (g/100 g protein)	
Methionine	1–1.4 (g/100 g protein)	
Valine	3.4–4.1 (g/100 g protein)	
Isoleucine	3.5–3.8 (g/100 g protein)	
Leucine	6.7–7.2 (g/100 g protein)	
Lysine	2.3–2.8 (g/100 g protein)	
Total essential amino acid	31.6–32.9 (g/100 g protein)	
Biological value	85.5–92.7 (g/100 g protein)	
Total phenolic content	1.2–2.94 mg/g	[1]
Chlorogenic acid	2.15–16.89 µg/g	[26,28]
Gallic acid	0.021–0.11 µg/g	
Ferulic acid	0.047–5.4 µg/g	
Protocatechuic acid	0.029–0.135 µg/g	
Caffeic acid	0.05–0.61 µg/g	
Hydroxybenzoic acid	0.011–1.46 µg/g	
p-Coumaric acid	0.035–2.41 µg/g	
Total flavonoid content	0.41–0.55 mg/g	
Quercitrin	4.78–184 µg/g	
Isovitexin	1.95–8.88 µg/g	
Afzelin	1.94–8.58 µg/g	
Isoquercitrin	1.8–5.08 µg/g	
Epicatechin	1.5–5.03 µg/g	
Vitexin	218.8–935.7 µg/g	
Hesperidin	1.83–4 µg/g	
Phloretin	0.04–0.165 µg/g	
Daidzein	0.03–0.06 µg/g	
Psoralen	0.008–0.014 µg/g	
Naringenin	0.005–0.227 µg/g	
(+)-catechin	0.001–3.13 µg/g	
Quercetin dihydrate	0.004–0.068 µg/g	
Rutin	0.17–11.9 µg/g	
Phlorizin	0.06–0.29 µg/g	
Glycitein	0.002–0.11 µg/g	

**Table 4:** Extraction medium used to recover TPC from barley.

Substrate	Extraction Medium (Solvent Type and Concentration)	TPC	Reference
Hulless barley	Methanol 80%	853.71–2331.93 µg FAE/g	[100]
Hulless barley	Methanol 80%	152.8–622 µg GAE/g	[101]
Hulless barley	Methanol 80%	1.12–1.33 mg GAE/g	[102]
Colored barley	Acidified methanol	2046–2402 µg GAE/g	[103]
Barley	Ethanol 50%	2.09–2.94 mg GAE/g	[1]
Barley	Acidified methanol	3.07–4.48 mg FAE/g	[90]
Barley	Methanol 80%	1.36–2.29 mg FAE/g	[104]
Barley	Methanol 80%	1.95–2.20 mg GAE/g	[89]
Barley	-	3.08–4.25 mg GAE/g	[105]
Barley straw	EtOH (20–100%)	32.6–57.7 mg GAE/g	[106]
Barley	Acetone 70%	0.30–1.99 mg/g	[107]
Barley	Ethanol 70%	0.30–1.52 mg/g	[107]
Barley	Methanol 70%	0.27–1.45 mg/g	[107]
Barley	Acetone–water–acetic acid (70:29.5:0.5 *v*/*v*/*v*)	0.80–1.30 mg/g	[108]
Hulless barley	Methanol 80%	0.42–5.4 mg GAE/g	[109]
Barley	Acidified methanol (1:100 *v*/*v*)	1.2–1.6 mg GAE/g	[110]
Barley	Methanol 95%	1.27 mg/g	[111]
Barley	Acetone 80%	1.44–1.76 mg GAE/g	[112]

**Table 5:** Primary uses and pharmaceutical potential of barley phytochemicals.

Category	Specific Phytochemical/Nutraceutical	Sources	Primary Uses	Pharmaceutical Potential	References
Phenolic Acids	Ferulic Acid	Bran and hull of barley grains	Strong antioxidant, protecting against cellular damage caused by free radicals. - Shields the skin from harmful UV radiation. - Anti-inflammatory responses. - Contributes to the structural integrity of plant cell walls, enhancing barley’s resistance to pathogens.	May reduce the risk of chronic diseases such as heart disease and certain types of cancer by combating inflammation and oxidative stress. - Could be used in skincare formulations to prevent UV-induced skin aging and damage. - Potential neuroprotective effects may help in the management of neurodegenerative disorders. - Offers the potential to improve gut health by contributing to the fiber content, which influences gut microbiota.	[113]
	p-Coumaric Acid	Whole grain, especially in the outer layers.	Antioxidant - Anti-inflammatory properties - Enhances liver health by supporting the body’s natural detoxification processes.	Potential to protect against liver disorders by promoting detoxification. - May help lower the risk of cardiovascular diseases by mitigating oxidative stress and inflammation. - Shows promise as a chemopreventive agent, particularly in reducing the risk of gastrointestinal cancers. - Could be incorporated into dietary supplements aimed at enhancing liver function and overall detoxification.	[114,115]
Tannins	Proanthocyanidins (Condensed Tannins)	Hull and seed coat.	Possesses strong antioxidant activity, which helps protect cells from oxidative stress. - Exhibits astringent properties, aiding in wound healing and reducing bleeding. - Demonstrates antimicrobial effects, which can inhibit the growth of harmful bacteria and support gut health. - Contributes to the bitterness and astringency of barley, influencing its use in brewing and food products.	Potential applications in wound care products due to their astringent and antimicrobial properties. - Could be developed into natural food preservatives or antimicrobial agents in food processing. - May play a role in cancer prevention by reducing oxidative stress and inhibiting cancer cell proliferation. - Useful in dietary supplements for promoting gut health and preventing infections.	[116]
Flavonoids	Catechins	Aleurone layer	Acts as a potent antioxidant, protecting against cellular damage from free radicals. - Supports cardiovascular health by improving blood circulation and reducing blood pressure. - Exhibits anticancer properties by inhibiting the proliferation of cancer cells and inducing apoptosis (programmed cell death). - May improve metabolic health by enhancing insulin sensitivity and regulating blood sugar levels.	Could reduce the risk of cardiovascular diseases by improving endothelial function and reducing oxidative stress. - Potential for use in cancer prevention and treatment strategies due to their ability to inhibit tumor growth and promote cancer cell death. - May be incorporated into dietary supplements aimed at improving metabolic health, particularly in managing diabetes and obesity. - Could be used in functional foods to enhance overall health and prevent chronic diseases.	[117]
Carotenoids	Lutein	Present in the endosperm and aleurone layers of barley grains	Promotes eye health by filtering harmful blue light and protecting against age-related macular degeneration (AMD). - Acts as an antioxidant, protecting cells from oxidative damage. - Supports skin health by protecting against UV-induced damage and improving overall skin appearance. - May contribute to cognitive health by reducing oxidative stress in the brain. - Plays a role in the photosynthetic processes of barley, helping the plant to capture light energy efficiently.	Potential as eye health supplements aimed at preventing AMD, cataracts, and other vision-related conditions. - Could be included in skincare products designed to protect against UV damage and promote skin health. - May support cognitive health and reduce the risk of neurodegenerative diseases by protecting brain cells from oxidative stress. - Potential for use in functional foods and dietary supplements targeting overall health and well-being.	[118]
	Zeaxanthin	Outer layers	Essential for eye health, particularly in preventing AMD and cataracts. - Acts as an antioxidant, protecting cells from oxidative damage and reducing the risk of chronic diseases. - Supports skin health by offering protection against UV radiation and maintaining skin integrity. - May improve overall eye function and visual performance, especially under conditions of high light exposure. - Contributes to the pigmentation and color of barley, influencing its appearance and nutritional quality.	Useful as eye health supplements aimed at preventing and managing conditions like AMD and cataracts. - Potential applications in skincare products for UV protection and antiaging benefits. - May help protect cognitive health by reducing oxidative stress and maintaining healthy brain function. - Could be incorporated into functional foods and supplements to promote overall health and protect against age-related decline.	[119,120]

**Table 6:** Future scope and novel uses of barley grains at a commercial scale.

Parts That Can Be Utilized	Sector/Product Type
Barley husks and other agricultural byproducts	Bioplastics and sustainable packaging as a sustainable alternative to petroleum-based plastics
Barley cellulose and hemicellulose	Biodegradable materials preparation
Barley-based agricultural byproducts	Biofuel preparation
Barley β-glucans, polyphenols, and flavonoids	Health-benefiting pharmaceuticals and functional ingredients
Barley extract	Cosmetics and skincare products
Whole barley or milling fraction	Barley-based enzymes, prebiotics, and beverages
Barley grains	Energy boosting drinks
Barley straw	Textile industries
Barley grains	Fortified food products and feed ingredients

## Data Availability

Not applicable.
